# Gut Microbiome of Children and Adolescents With Primary Sclerosing Cholangitis in Association With Ulcerative Colitis

**DOI:** 10.3389/fimmu.2020.598152

**Published:** 2021-02-05

**Authors:** Ramon V. Cortez, Luana N. Moreira, Marina Padilha, Mariana D. Bibas, Ricardo K. Toma, Gilda Porta, Carla R. Taddei

**Affiliations:** ^1^ Department of Clinical and Toxicological Analysis, School of Pharmaceutical Sciences Universidade de Sao Paulo (USP), São Paulo, Brazil; ^2^ School of Pharmaceutical Sciences, Universidade de Sao Paulo, São Paulo, Brazil; ^3^ Department of Gastroenterology and Hepatology, Child Institute, ICR-HC/FM, Universidade de Sao Paulo (USP), São Paulo, Brazil; ^4^ Department of Hepatology and Liver Transplantation, Hospital Sirio Libanes/Hospital Municipal Infantil Menino Jesus, São Paulo, Brazil; ^5^ School of Arts, Science and Humanities, University of Sao Paulo, São Paulo, Brazil

**Keywords:** primary sclerosing cholangitis, ulcerative colitis, gut microbiome, 16S rRNA, dysbiosis

## Abstract

Few studies reported the relation of intestinal microbiome composition and diversity in pediatric patients with primary sclerosing cholangitis (PSC) and ulcerative colitis (UC). In this cross-sectional study, we selected patients younger than 19 years old from the pediatric gastroenterology and hepatology outpatient clinic of a tertiary hospital to describe the intestinal microbiome of pediatric patients with PSC associated or not to UC. Patients were divided in PSC, PSC+UC, and UC diagnosis. A stool sample was collected from each patient (n=30) and from a healthy relative/neighbor (n=23). The microbiome composition was assessed using MiSeq (Illumina) platform. Differences in microbial composition were found between PSC and PSC+UC groups. The relative abundance of *Veillonella* and *Megasphaera* genera were increased depending on patients’ age at diagnosis. *Veillonella* was also increased in patients who were in an active status of the disease. Both genera were positively correlated to total bilirubin and gamma-glutamyl transferase. As a conclusion, the disease, the age and the disease activity status seem to influence the intestinal microbiome, highlighting the difference of intestinal microbiome profile for patients depending on age at diagnosis. We also showed an increase of *Veillonella* in patients with PSC and PSC+UC, and a positive correlation of dysbiosis and higher gamma-glutamyl transferase and total bilirubin in PSC+UC patients. Our findings are promising in the diagnosis, prognosis, and future therapeutic perspectives for PSC patients.

## Introduction

Primary sclerosing cholangitis (PSC), a chronic inflammatory disorder that affects the hepatobiliary system, is characterized by an inflammatory process, leading to progressive fibrosis of intra- and/or extrahepatic bile ducts (1). Periductular fibrosis (“onion skin fibrosis”) is the hallmark for the diagnosis of PSC. The diagnosis of PSC is based on endoscopic retrograde cholangiopancreatography or magnetic resonance cholangiopancreatography. Clinically, PSC can progress to cholestasis, cholangitis, cirrhosis, and hepatic failure, with associated complications (2, 3) usually affecting the entire biliary tree (4).

Although PSC is more frequently reported in adults (5, 6), it can start at any age. In pediatric patients, the age at diagnosis is usually around 10 to 16 years of age (7, 8). The symptoms, progression and laboratory markers of the disease differ between pediatric and adults patients, which might explain distinct outcomes according to the age (4).

There is a strong association between PSC and inflammatory bowel disease (IBD) (9); recently, Deneau et al. (1) found that 76% of 781 children in the Pediatric PSC consortium had concomitant IBD, mostly ulcerative colitis (UC) or IBD-unclassified (83%). Additionally, Lee et al. (10) reported that 71% of children had PSC and IBD, concomitantly.

The etiology and pathogenesis of PSC and the causes of its association with UC remain unknown. The initial event and the mechanisms responsible for progressive changes in PSC appear to be due to an immunologically mediated process (11). In addition, studies were carried out to explain the peculiar factors of PSC, mainly the strong association with UC. These studies suggest that the intestinal microbiota could be a potential link (9, 12). In this situation, the microbiota would favor intestinal inflammation and enterohepatic circulation of bacteria, lymphocytes, or pro-inflammatory molecules derived from the intestine. Thus, this communication between the intestine and the liver could lead to portal and biliary inflammation in genetically predisposed individuals. This concept forms the basis for the so-called microbiota hypothesis of PSC (13–15), resulting in tissue destruction concomitant with an innate immune response to intestinal microbiota antigens, which activate an abnormal immune response in predisposed individuals, as well as in UC (12, 16).

To our knowledge, no previous study has reported the intestinal microbiome composition in patients under 10 years of age diagnosed with PSC or PSC with concomitant UC. In this study, we evaluated the intestinal microbiome composition of children and adolescents diagnosed with PSC, UC, and those with PSC with concomitant UC, compared to healthy participants.

## Materials and Methods

### Study Population

We performed a prospective study enrolling children aged 3 to 19 years between May 2016 and June 2017, undergoing evaluation for PSC, UC, or PSC with concomitant UC (PSC+UC), from the pediatric Hepatology and Gastroenterology outpatient clinic of Child Institute - Hospital das Clinicas da Faculdade de Medicina de Sao Paulo (ICR – HCFM), Brazil. Additionally, healthy controls who were siblings or close relatives aged 2 to 21 years living in the same house or nearby were enrolled (**Table 1**). The diagnosis of PSC was made based on characteristic bile duct changes with multifocal structures and segmental dilatation by magnetic resonance cholangiopancreatography (MRCP), clinical presentation, cholestatic biochemical profile, and no evidence of secondary sclerosing cholangitis. Patients with autoimmune sclerosing cholangitis and small ducts were excluded by clinical and/or liver biopsy. The diagnosis of UC was made according to established clinical, biochemical, radiologic, endoscopic, and histologic criteria using the revised Porto criteria (17). The disease activity was performed using the Pediatric Ulcerative Colitis Activity Index (PUCAI) (18) clinical score, blood tests and endoscopic appearance. This study was approved by the ethical committee of ICR-HCFM (CAAE 33876620.0.0000.0068), and signed informed consent was obtained from all subjects who provided specimens and their parents.

**Table 1:** General characteristics from groups.

		Ulcerative Colitis (UC)			Primary Sclerosing Cholangitis (PSC)			PSC+UC		
		Cases	Controls	*P*	Cases	Controls	*P*	Cases	Controls	*P*
**Age (years)** ^a^		12 (3–17)	8 (2–15)	0.036*	14 (9–16)	14 (7–21)	0.92	12 (6–16)	11 (3–17)	0.72
**Sex** ^b^	Male	8 (66.7%)	5 (50%)	0.43	5 (45.5%)	6 (85.7%)	0.11	4 (57.1%)	5 (83.3%)	0.32
	Female	4 (33.3%)	5 (50%)		6 (54.5%)	1 (14.3%)		3 (42.9%)	1 (16.7%)	
**Type of delivery** ^b^	Vaginal	7 (58.3%)	4 (40%)	0.39	4 (36.4%)	1 (16.7%)	0.40	4 (57.1%)	4 (66.7%)	0.72
	Cesarean	5 (41.7%)	6 (60%)		7 (63.6%)	5 (83.3%)		3 (42.9%)	2 (33.3%)	
**Breastfeeding time** ^b^	> 4 months	10 (83.3%)	10 (100%)	0.99	9 (81.8%)	3 (50%)	0.18	7 (100%)	4 (66.7%)	0.99
	< 4 months	2 (16.7%)	0		2 (18.2%)	3 (50%)		0	2 (33.3%)	

^a^Mean (minimum-maximum); ^b^number (percentage); P values are based on Generalized linear model (GzLM) with ^a^linear distribution and ^b^ordinal logistic distribution; *Significant when P ≤ 0.05.

### Sample Collection and DNA Isolation

Fecal samples were collected from each child enrolled in this study. Each participant was instructed to collect one stool sample at home using a dry and sterile stool collector and keep it in a freezer (−20°C) until the medical appointment, some hours later. Samples were transported in an ice-filled polystyrene container (previously supplied to the patient). At the hospital, the samples were kept at −80°C, until further analyses. DNA was obtained from stool samples using the QIAamp DNA Stool Mini Kit (Qiagen), according to the manufacturer’s protocol.

### 16S rRNA Gene Sequence Processing

Amplification of the V4 region of the 16S rRNA gene, library preparation and sequencing steps were performed, on a single run, as previously described (19). Raw read files were analyzed using QIIME software (20). All reads lower than 400 base pairs were discarded. Chimeric sequences were excluded using usearch61 (21). Based on 99% similarity, the remaining sequences were compared against Silva (22) version 128 and grouped into operational taxonomic units (OTUs). Nucleic acid sequences are available at the Sequence Read Archive (SRA) under accession number PRJNA610934.

### Data Analysis

Species richness and alpha diversity were estimated by Chao1 (23), Shannon (24), Simpson (25) and Observed OTUs indices. Principal coordinate analyses (PCoA) were generated based on weighted and unweighted UniFrac phylogenetic distance matrices to observe differences in beta diversity between groups (26, 27). Bray-Curtis distance was used to calculate the distances between disease participants and their respective control participants for “active” or “remission” disease status at the moment of enrollment. To observe differences related to age at sample collection/diagnosis, after the microbial analysis of the disease groups versus controls, we subsampled cases in patients younger and older than 10 years old.

### Statistical Analyses

Statistical analysis was performed in SPSS version 22 and R (R version 3.4.3, Vienna, Austria), using the phyloSeq (28), vegan (29) and ggplot2 (30) packages. The generalized linear model (GzLM) was used to compare the groups to the consecutive controls in relation to descriptive data and to compare PSC, UC, and PSC+UC group in relation to clinical data through linear and ordinal logistic distribution. This model was also used to evaluate the effect of the independent variables (groups) on the dependent variables (for alpha diversity indices, using gamma distribution, and for bacterial phyla and genera relative abundance using linear distribution). To observe differences in beta diversity between groups, PERMANOVA was performed using the adonis function for both weighted and unweighted UniFrac distances. For each variable, 999 permutations were made. Mann-Whitney test was used to compare the distributions of the distances between disease and control participants for “active” or “remission” status. The influence of age on the bacterial relative abundance and diversity was evaluated by sorting the participants in two groups: under and above 10 years old, considering the clinical relevance of the early and late onset diagnosis. One-tailed Pearson’s correlation was used to observe the correlation between clinical data and bacterial genera. For all analyses, the level of significance considered was *P* ≤ 0.05.

## Results

### General Characteristics and Clinical Data

Thirty patients were included in this study, which were allocated in the PSC group (n=11), UC group (n=12), and PSC+UC group (n=7); additionally, 23 healthy children/adolescents were included to represent the control group. The general characteristics and clinical data at collection are described in **Tables 1** and **2**, respectively. Overall, no differences were found between groups regarding general and clinical characteristics, except for the age in the UC group (12 vs 8 years of age in case and control groups, respectively; **Table 1**). Values of gamma-glutamyl transferase (GGT), reactive C protein (RCP), total bilirubin and albumin from patients were monitored during all follow-up (**Table 2** and **Supplementary Table 1**). At collection, GGT values were significantly increased in the PSC+UC group compared to the UC and PSC groups, and albumin values were significantly increased in the UC group compared to the PSC group (**Table 2**). In addition, most of the UC patients were in remission of the disease, and most of the PSC+UC patients were in activity (**Table 2**). Moreover, only one participant used antibiotics in the last 12 months prior to collection.

**Table 2:** Clinical data from disease groups.

		Ulcerative Colitis (UC)	Primary Sclerosing Cholangitis (PSC)	PSC+UC	*P*
**Age of diagnosis (years)** ^a^		7 (0–12)	10 (0–14)	7 (3–12)	0.14
**Reactive C protein (mg/L)** ^b^	< 0.3	6 (50%)	3 (30%)	2 (28.6%)	0.53
	> 0.3	6 (50%)	7 (70%)	5 (71.4%)	
**Albumin (g/dl)** ^c^		4.44 (0.32)	3.97 (0.67)	4.10 (0.35)	0.048*
**Gamma-Glutamyl Transferase (U/L)** ^c^		15.08 (3.91)	109.63 (155.31)	413.00 (430,90)	<0.001*
**Total Bilirubin (mg/dl)** ^c^		0.54 (0.31)	2.04 (3.62)	0.99 (1.04)	0.26
**Leukocytes (×10^9^/L)** ^c^		7.63 (2.97)	6.32 (2.04)	6.47 (1.41)	0.32
**Disease state** ^b^	Active	3 (25%)	4 (36.4%)	5 (71.4%)	0.16
	Remission	9 (75%)	7 (63.6%)	2 (28.6%)	

^a^Mean (minimum-maximum); ^b^number (percentage); ^c^mean (standard deviation); P values are based on Generalized linear model (GzLM) with ^a,c^linear distribution and ^b^ordinal logistic distribution; *Significant when P ≤ 0.05.

### Cases vs Controls

**Figure 1:**
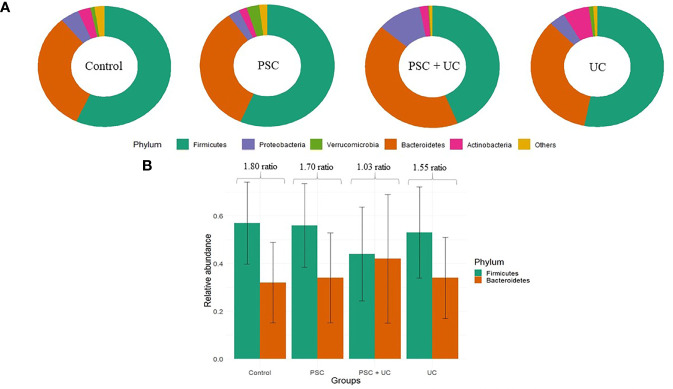
Relative abundance of the main phyla observed in this study. **(A)** Main phyla in patients according to groups. **(B)** Barplot of the Firmicutes/Bacteroidetes ratio for each group. PSC, Primary Sclerosing Cholangitis diagnosed patients; UC, Ulcerative Colitis diagnosed patients.

Firmicutes and Bacteroidetes were the predominant phyla found in all groups (**Supplementary Table 2**). However, the relative abundance of Firmicutes was higher in the control, PSC and UC groups, whereas the relative abundance of Bacteroidetes was higher in the PSC+UC group. The levels of Proteobacteria observed in the PSC+UC group were much higher than those in the other groups (**Figure 1A**), but none of these results reached statistical significance. In addition, we observed that the Firmicutes/Bacteroidetes (F/B) ratio was well diminished in the PSC+UC group compared to the other groups (**Figure 1B**).

In general, the most abundant genera observed were *Bacteroides*, *Prevotella 9*, *Lachnospiraceae NK4A136 group, Ruminococcaceae UCG 002*, *Veillonella*, and *Megasphaera* (**Supplementary Figure l** and **Supplementary Table 3**). The genus *Bacteroides* was predominant in all groups, except for the PSC+UC group, where *Prevotella 9* was the most abundant. In the comparison between cases and controls, *Veillonella* (*P*=0.002) was significantly more abundant in the PSC+UC group. Other genera, such as *Eubacterium coprostanoligenes group* (*P*=0.22), *Ruminococcaceae UCG 002* (*P*=0.06) and *Christensenellaceae R7 group* (*P*=0.08), were significantly higher in the control group, but the results were not maintained in *post hoc* test. The same occurred for genera more abundant in the UC group, *Acidaminococcus* (*P*=0.17), and in the PSC group, *Streptococcus* (*P*=0.07) and *Megasphaera* (*P*=0.16). There was a positive correlation between the abundance of *Megasphaera* and higher values of GGT (*P*=0.032) and the abundance of *Veillonella* and higher values of bilirubin (*P*=0.015) in the PSC+UC group (**Supplementary Table 4**).

### Microbiome Analysis

To verify the hypothesis of different microbial composition in PSC pediatric patients according to age at the diagnosis, after the microbial analysis of the diseases groups versus controls, we subsampled the cases in younger than 10 years (<10 years) and older than 10 years old (>10 years) at the sample collection and/or diagnosis.

### Microbiome of Children Under 10 Years of Age

Children under 10 years of age were subsampled according to age at sample collection; therefore, we subsampled them at <10 years of age at diagnosis - PSC group (n=3), UC group (n=6), PSC+UC group (n=3), and Control Group (n=11). The predominant phyla were Firmicutes and Bacteroidetes (**Supplementary Table 5**), and Firmicutes was more abundant in the PSC group and Bacteroidetes was more prevalent in the UC group. The relative abundances of Proteobacteria and Actinobacteria were significantly higher in the PSC+UC (*P*=0.01) and UC (*P*=0.01) groups, compared to the control group.

**Figure 2:**
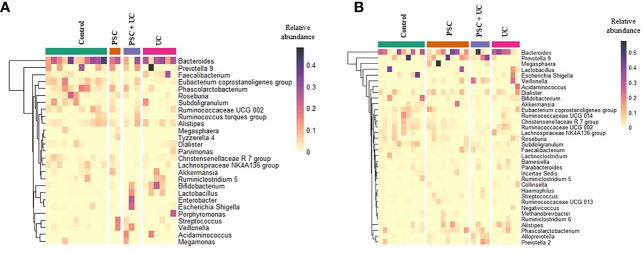
Relative abundance heatmap of the most abundant bacterial genera identified in fecal samples. **(A)** Patients and control groups under than 10 years of age of diagnosis and **(B)** over 10 years. The rows present the genera identified with maximum relative abundance higher than 0.05. Column represents the samples from patients and control groups. PSC, Primary Sclerosing Cholangitis; UC, Ulcerative Colitis.

**Figure 3:**
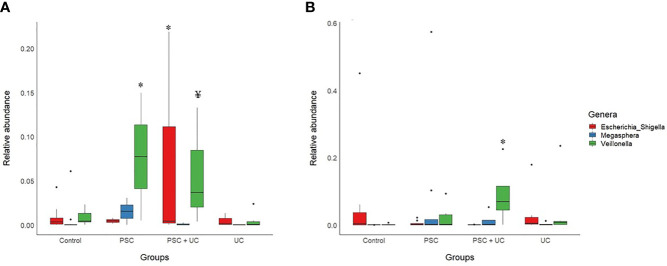
Barplot of statistically significant bacterial genera, according to age. **(A)** Patients under than 10 years of age; **(B)** Patients over than 10 years of age. PSC, Primary Sclerosing Cholangitis; UC, Ulcerative Colitis; * indicates statistical significance compared to the control group (after Sidak’s *post hoc* test); ¥ Significance in comparison to the control group was not maintained in *post hoc* test.

The most abundant bacterial genus found in children <10 years old was *Bacteroides* (**Figure 2A** and **Supplementary Table 6**), which was more prevalent in the UC group and less prevalent in the PSC+UC group, but the results were not statistically significant. Interestingly, the relative abundance of *Bifidobacterium* was significantly higher in the UC group than in controls (*P*=0.04). The abundance of *Streptococcus* in the PSC group was statistically higher than that in the control group (*P*=0.001). In addition, the abundance of the genus *Veillonella* was significantly greater in the PSC and PSC+UC groups than in controls (*P*=0.03 and *P*=0.01, respectively), and *Escherichia-Shige*lla showed significantly higher values in the PSC+UC group than in controls (*P*=0.04) (**Figure 3A**).

### Microbiome of Children and Adolescents Over 10 Years of Age

The samples divided in this category were unable to be subsampled according to age at diagnosis, since the sample size was too small at this analysis stage. Thus, we subsampled according to age at collection - PSC group (n=8), UC group (n=6), PSC+UC group (n=4), and Control Group (n=10). The predominant phyla in patients over 10 years of age were Firmicutes and Bacteroidetes (**Supplementary Table 7**), Firmicutes more prevalent in the UC group and Bacteroidetes more prevalent in the PSC+UC group. The predominant genus in these patients was *Bacteroides* (**Figure 2B** and **Supplementary Table 8**), more prevalent in the UC group. We observed a tendency for higher abundance of *Prevotella 9* in the PSC+UC group compared to the control group (*P*=0.06), while *Lactobacillus* was decreased in the same group. Moreover, the abundance of the genus *Veillonella* was significantly higher in the PSC+UC group than in the control group (*P*=0.02) (**Figure 3B**).

### Alpha Diversity

**Figure 4:**
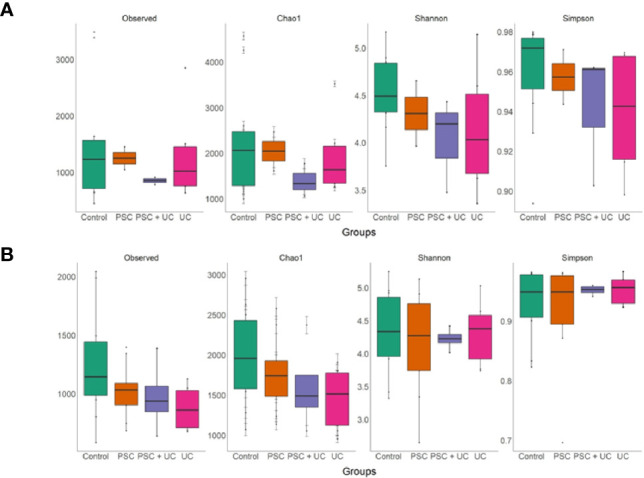
Richness and alpha diversity values of samples, by groups. **(A)** Samples from patient and control groups under 10 years of age and **(B)** over 10 years of age. The richness and alpha diversity are measured by Observed OTUs, Chao1, Shannon, and Simpson indices. The box-plot is representing the interquartile range (IQR) and the line inside represents the median. The generalized linear model was performed to compare the values between the groups.

Alpha diversity of children <10 years old showed higher levels of richness (Chao1), diversity (Shannon) and observed OTUs in the control group (**Figure 4A** and **Supplementary Table 9**), and Chao1 index was significantly lower in the PSC+UC group compared to controls (*P*=0.05). In contrast, the PSC group presented higher values of Simpson. In patients over 10 years of age (**Figure 4B** and **Supplementary Table 10**), a higher richness (Chao1) and observed OTUs was identified in controls, but the results were not statistically significant. In addition, we observed that the mean values for Shannon and Simpson were similar in controls and cases in this age group, differently than children < 10 years of age (**Figure 4**).

### Disease Status—Active vs Remission/Controlled Disease

**Figure 5:**
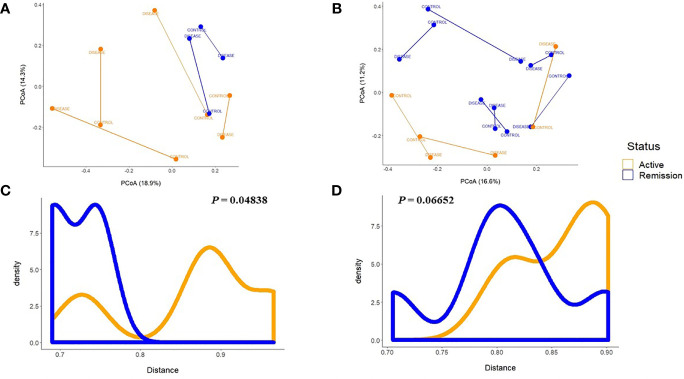
Principal Coordinate Analyses plots of Bray-Curtis distance between each patient and the respective control. **(A)** According to the disease status of patients in the PSC + UC or **(B)** UC groups, and according to the active or remission disease status shows significant differences for the PSC + UC group **(C)**, but not for the UC group **(D)** by the Mann–Whitney test. UC, Ulcerative Colitis group; PSC + UC, Primary Sclerosing Cholangitis with concomitant Ulcerative Colitis.

To evaluate the influence of active or remission/controlled disease status on the intestinal microbiome, we explored the distances between patients and their respective healthy control according to the disease status (**Figures 5A, B**). In the PSC+UC group (**Figure 5C**), the distances between patients in active disease status and their respective healthy control were significantly higher than the distances between patients in remission disease status from their controls (*P*=0.048). We observed a similar tendency for the UC group (**Figure 5D**); however, it was not statistically significant (*P*=0.066).

Subsequent analysis, independent of age and disease groups – remission/controlled patients (n=18) and patients in activity (n=12), showed a significant higher relative abundance of *Ruminoclostridium 5* (*P*=0.02) and *Ruminococcaceae UCG 002* (*P*=0.047) in patients in remission/controlled disease compared to patients with active disease (**Supplementaty Table 11**). In addition, *Veillonella* was increased in patients with active disease (*P*=0.01), and *Escherichia-Shigella* tended to increase in this group (*P*=0.06).

## Discussion

The influence of the intestinal microbiome on PSC development has been described in recent years. Indeed, changes in microbial composition have been observed in PSC/UC studies with adult patients (31), suggesting the role of the intestinal microbiome in the course of the disease and confirming the previous hypothesis of the “PSC microbiome” (12). In this sense, it is essential to describe this relationship in either pediatric or adolescent patients.

Firmicutes is the major phyla in human intestinal microbiome, depicting approximately 60%–65% of microbiome diversity (32). Bacteroidetes is the second most abundant phylum, comprising genera involved in degradation of soluble carbohydrates in intestinal lumen. The Firmicutes/Bacteroidetes ratio has already been described as a marker of eubiosis/dysbiosis in obese and diabetic patients (33, 34). The increase in Firmicutes abundance and decrease in Bacteroidetes abundance was related to an imbalance in intestinal microbiome composition and consequently metabolic disorders. Here, we describe new abundance profiles for these phyla; there was no difference in the proportion of these phyla in patients with the association of PSC and UC. The increases in the abundance of *Prevotella 9* and *Bacteroides*, members of the Bacteroidetes phylum, in the PSC+UC group were different than the other groups. In addition to increased Bacteroidetes abundance, the PSC+UC group showed the highest levels of Proteobacteria, which might explain the decrease in Firmicutes abundance.

IBD is a well-characterized intestinal disease (35) associated with microbiome dysbiosis in both children and adult patients (36). Montreal classification (37) divided IBD occurrence between pediatric (<17 years old) and adult disease onset. The Paris Classification divided the pediatric disease into late onset (LO, >10 years old) and early onset (EO, <10 years old) based on age at diagnosis. In this way, very early onset (VEO) was proposed to define children under 6 years of age at diagnosis. It is already known that in IBD patients, the severity of the disease increases with age; however, in VEO patients, there are more complications in the disease course since it starts earlier (37).

Iwasawa et al. (38) recently studied the microbiome composition of saliva from children with PSC and UC, showing different results in the salivary microbiome between the PSC group and healthy controls. The definition of pediatric onset for PSC was proposed by the authors. Here, we propose the use of early onset for patients younger than 10 years of age and late onset for those older than 10 years of age, including PSC+UC diagnosis, since there is a distinct microbiome pattern between these groups. Intestinal dysbiosis in pediatric patients might be considered a possible indicator for disease outcomes (38).

PSC patients showed a distinct pattern of fecal microbiome according to the age of diagnosis/sample collection. The abundance of *Veillonella* was significantly increased in the fecal microbiome of patients with PSC and PSC+UC in both early- and late-onset patients compared to the control and UC groups. However, in early-onset patients, the abundance of *Veillonella* was remarkably increased, followed by an increase in *Escherichia-Shigella* abundance only in PSC+UC patients. Interestingly, *Megasphaera* increased in abundance in late-onset patients only in the PSC group. Several previous studies reported an increased in the abundance of *Megasphaera* and *Veillonella* in a study of older children (8) and adults (5, 39). *Veillonella* and *Megasphaera* are gram negative anaerobic roads, belonging to the *Veillonellaceae* family and Firmicutes Phylum. They are usually described as members of mouth and gut human microbiome (40, 41), and rarely described in human infections (42). Little is known about the role of these genera in human intestinal microbiome equilibrium.

In our study, we found a positive correlation between the abundance of *Veillonella* and higher bilirubin values and the abundance of *Megasphaera* and higher GGT values, suggesting the role of the microbiome in disease severity. We were unable to correlate these findings to early or late onset due to our small sample size in each group. Nakamoto et al. recently showed a cooperative relationship between pathobionts in a PSC-UC gnotobiotic animal model assay (43). Their findings suggested bacterial translocation and an association with increases in the abundance of *Klebsiella* and hepatobiliary diseases. They were not able to detect *Veillonella* in the studied human cohort; however, they included adult patients. We could suggest that *Veillonella* might be related to PSC outcome in children and adolescent patients, since this genus was already correlated to the production of amine oxidases and contributed to the manifestation of PSC+UC *via* aberrant lymphocyte tracking between the bowel and liver (44).

Here, we report a distinct fecal microbiome pattern in PSC and PSC+UC patients in early onset compared to those in late onset. Since the abundance of *Veillonella* in children with pediatric PSC and PSC+UC was higher, this bacterium might be related to a biomarker of PSC in younger children associated with clinical laboratory values, including GGT and bilirubin (1).

Clinically, PSC patients with higher GGT and bilirubin values, among others, are generally related to worse outcomes (1). In this transversal pilot study, we showed that PSC associated with UC is related to intestinal microbiome dysbiosis in younger children and positively correlated with high GGT and bilirubin values. Despite our small sample size, the worse outcomes at 6 and 12 months of follow-up were for patients with high values of GGT and bilirubin at collection (**Supplementary Table 1**). However, a longitudinal study is necessary to establish intestinal dysbiosis and worse outcomes.

Interestingly, *Veillonella* was also significantly increased in the intestinal microbiome in patients with active disease at sample collection. The significant distance between microbial community structures in both remission and active disease groups and the evidence of the increased *Veillonella* in the active disease group reinforce the evidence of a role of the intestinal microbiome in the course of the disease, as discussed above.

We note the small sample size of children in each group, particularly children under 10 years old, as a limitation of the present study, which weakens the final results. In addition, the lack of a complete longitudinal follow-up is another limitation. The inclusion of more participants could increase the strength of data on microbial abundance variation and bacterial genera and lead to more significant results. The strengths of this study are the control group with family members or relatives, avoiding diet and environmental influences on microbiome results, and the inclusion of young children with PSC+UC and PSC diagnosis.

In conclusion, we described here the intestinal microbiome of children and adolescents with PSC and/or associated UC, highlighting the difference in the intestinal microbiome profile for early- and late-onset patients. Our work shows the relationship of microbiota in cases of active disease and especially dysbiosis in patients with an association of PSC and UC. This dysbiosis might be related to the different pathophysiology of the disease in children. To our knowledge, this is the first study describing the intestinal microbiome in children under 10 years old with PSC and PSC+UC. Since we could verify the hypothesis of different microbial compositions in PSC pediatric patients according to age at diagnosis, we are proposing the categorization of early and late PSC and PSC+UC onset according to the age at diagnosis. We also showed an increase in *Veillonella* in patients with PSC and PSC+UC and a positive correlation between higher GGT values and higher *Veillonella* abundance, suggesting the potential use of this bacterial genus as a biomarker of PSC. These findings could open new possibilities for diagnosis and prognosis and future therapeutic options in pediatric PSC care.

## Data Availability Statement

The data sets presented in this study can be found in online repositories. The name of the repository and accession number can be found here: https://www.ncbi.nlm.nih.gov/genbank/, PRJNA610934.

## Ethics Statement

The studies involving human participants were reviewed and approved by Ethical committee of ICR – HCFM (CAAE 33876620.0.0000.0068). Written informed consent to participate in this study was provided by the participants’ legal guardian/next of kin.

## Author Contributions

RC: sample preparing for sequencing, sequencing and data analyses, draft the manuscript, bioinformatic analyses and submission of samples to GenBank. LM: sample preparing for sequencing and sequencing. MP: bioinformatic analyses. MB. enrollment of patients and sample collection. RT: study design, draft the manuscript, enrollment of patients and data interpretation. GP: study design and critical revision of the manuscript for important intellectual content. CT: Acquisition of the financial support for the project leading to this publication, draft the manuscript, data analyses and interpretation. All authors have approved the final draft of the manuscript for submission.

## Funding

This research was supported by fellowships from FAPESP N. 2015/13059-9 to CT, and the Coordenação de Aperfeiçoamento de Pessoal de Nível Superior (CAPES)-Finance Code 001. The funding agencies had no role in the design, preparation, review, or approval of this study.

## Conflict of Interest

The authors declare that the research was conducted in the absence of any commercial or financial relationships that could be construed as a potential conflict of interest. 

The handling editor declared a shared affiliation, though no other collaboration, with the authors.
