# Supplementary material for: Gut Microbiome of Children and Adolescents With Primary Sclerosing Cholangitis in Association With Ulcerative Colitis
Source: Front Immunol. 2021 Feb 5;11:598152. doi: 10.3389/fimmu.2020.598152 (PMC7893080; doi:10.3389/fimmu.2020.598152)
Supplement: Supplementary file 1 [file Image_1.pdf]

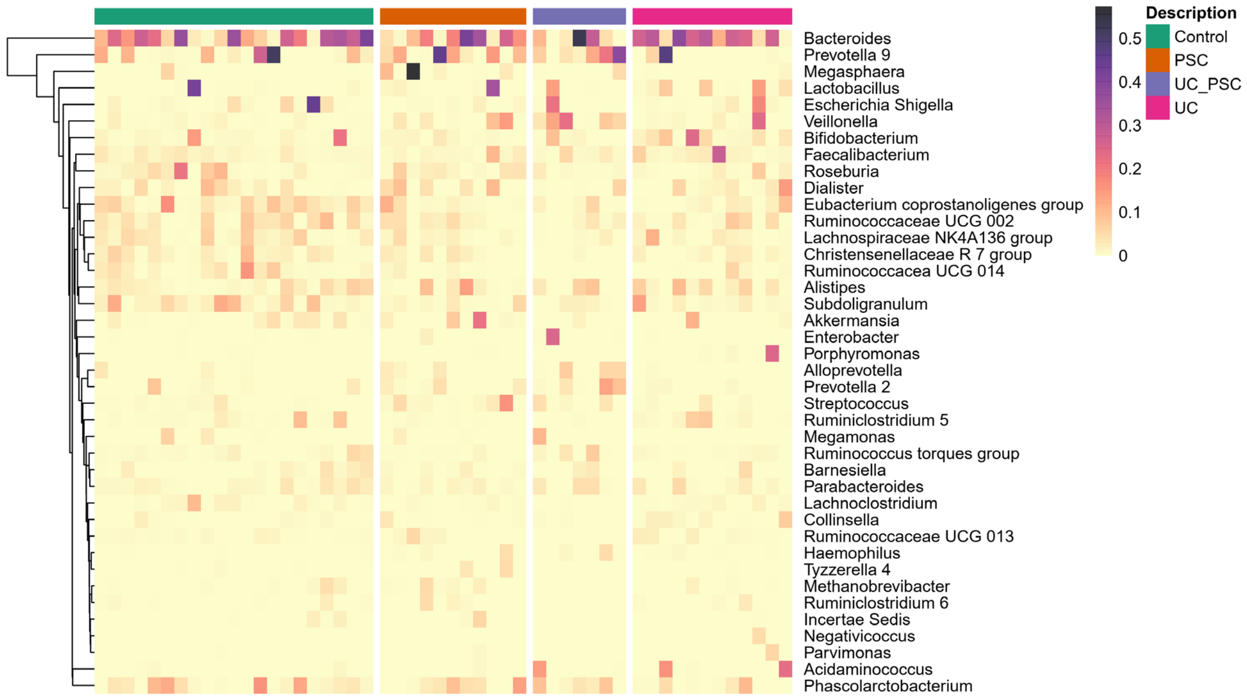

Supplementary Figure 1. Relative abundance heatmap of the most abundant bacterial genera identified in fecal samples.

Legend: The rows present the genera identified with maximum relative abundance higher than 0.05. Column represents the samples from patients and control groups; PSC = Primary Sclerosing Cholangitis; UC = Ulcerative Colitis.
