# Supplementary material for: Gut Microbiome of Children and Adolescents With Primary Sclerosing Cholangitis in Association With Ulcerative Colitis
Source: Front Immunol. 2021 Feb 5;11:598152. doi: 10.3389/fimmu.2020.598152 (PMC7893080; doi:10.3389/fimmu.2020.598152)
Supplement: Supplementary file 2 [file Table_1.docx]

| **Supplementary Table 1.** Clinical follow-up (6 months and 1 year) per patient. | | | | | | | | |
| --- | --- | --- | --- | --- | --- | --- | --- | --- |
| **ID** | **Group** | **Disease State** | | | **Genera abundance (%)** | | **Clinical Data** | |
|  |  | **Inclusion** | **Follow-up**  **6 months** | **Follow-up**  **1 year** | ***Megasphaera*** | ***Veillonella*** | **TB^a^** | **GGT^b^** |
| 1 | Early onset  UC | Active | Remission | Remission | 0.00 | 2.40 | 0.54 | 15 |
| 5 |  | Active | Active | Remission | 0.00 | 0.00 | 0.15 | 16 |
| 7 |  | Remission | Remission | Remission | 0.00 | 0.50 | 0.14 | 19 |
| 20 |  | Remission | Active | Remission | 0.00 | 0.10 | 0.50 | 15 |
| 40 |  | Remission | Remission | Remission | 0.00 | 0.00 | -- | 19 |
| 53 |  | Remission | Remission | Remission | 0.00 | 0.00 | 0.18 | 9 |
| 25 | Late onset UC | Remission | Remission | Remission | 0.30 | 0.80 | 0.59 | 14 |
| 14 |  | Active | Remission | Remission | 0.00 | 23.20 | 1.02 | 9 |
| 13 |  | Remission | Remission | Remission | 1.30 | 0.00 | 0.58 | 12 |
| 23 |  | Remission | Remission | Remission | 0.00 | 0.60 | 0.84 | 13 |
| 11 |  | Remission | Remission | Discharged | 0.00 | 0.10 | 1.02 | 20 |
| 39 |  | Remission | Remission | Remission | 0.00 | 1.30 | 0.45 | 20 |
| 3 | Early onset PSC+UC | Active | Remission PSC / UC | Remission PSC / UC | 0.00 | 13.00 | 0.35 | 28 |
| 9 |  | Remission | Active PSC / Remission UC | Active PSC / Remission UC | 0.00 | 0.40 | 0.31 | 137 |
| 27 |  | Remission | Remission PSC / UC | Remission PSC / UC | 0.30 | 3.70 | 0.27 | 28 |
| 19 | Late onset PSC+UC | Active | Discharged | Discharged | 0.00 | 22.00 | 3.22 | 859 |
| 29 |  | Active | Active PSC / Remission UC | Active PSC / Remission UC | 5.30 | 6.20 | 1.1 | 1138 |
| 34 |  | Active | Liver transplant | Liver transplant | 0.20 | 8.00 | 1.06 | 283 |
| 45 |  | Active | Active PSC / Remission UC | Active PSC / Remission UC | 0.10 | 0.10 | 0.65 | 418 |
| 46 | Early onset PSC | Remission | Active | Active | 2.90 | 15.50 | 1.68 | 135 |
| 58 |  | Remission | Remission | Active | 0.00 | 0.50 | 0.28 | 18 |
| 51 |  | Remission | Active | Active | 0.00 | 3.50 | 0.63 | 211 |
| 31 | Late onset PSC | Active | Remission | Remission | 0.20 | 0.30 | 0.82 | 544 |
| 49 |  | Remission | Remission | Remission | 56.90 | 0.10 | 0.82 | 14 |
| 33 |  | Remission | Remission | Remission | 0.00 | 3.00 | 1.58 | 62 |
| 36 |  | Active | Liver transplant | Liver transplant | 0.10 | 9.20 | 12.87 | 54 |
| 42 |  | Remission | Remission | Remission | 1.60 | 0.00 | 0.29 | 49 |
| 37 |  | Active | Remission | Remission | 9.70 | 0.20 | 1.62 | 46 |
| 56 |  | Remission | Remission | Remission | 0.20 | 0.00 | 0.76 | 24 |
| 48 |  | Active | Remission | Remission | 0.00 | 0.10 | 1.17 | 52 |
| ^a^ Total Bilirubin (mg/dL); ^b^ Gamma-Glutamyl Transferase (U/L); -- Missing data; **UC =** Ulcerative Colitis; **PSC =** Primary Sclerosing Cholangitis; **PSC + UC** = Presence of both diseases. | | | | | | | | |
