# Supplementary material for: Gut Microbiome of Children and Adolescents With Primary Sclerosing Cholangitis in Association With Ulcerative Colitis
Source: Front Immunol. 2021 Feb 5;11:598152. doi: 10.3389/fimmu.2020.598152 (PMC7893080; doi:10.3389/fimmu.2020.598152)
Supplement: Supplementary file 3 [file Table_2.docx]

| **Supplementary Table 2**. Relative abundance of the main phyla observed in controls and case groups. | | | | | | | |
| --- | --- | --- | --- | --- | --- | --- | --- |
| **Groups**  **Phyla** | **Control** | **UC** | | **PSC + UC** | | **PSC** | |
|  | Mean (SD) | Mean (SD) | *P ^a^* | Mean (SD) | *P ^a^* | Mean (SD) | *P ^a^* |
| **Firmicutes** | 57.28 (17.29) | 53.35 (17.62) | 0.53 | 43.87 (19.44) | 0.07 | 56.77 (19.00) | 0.94 |
| **Bacteroidetes** | 31.89 (16.90) | 34.42 (19.01) | 0.70 | 42.59 (26.96) | 0.18 | 33.45 (16.86) | 0.82 |
| **Proteobacteria** | 4.43 (9.92) | 3.80 (5.17) | 0.86 | 10.46 (20.62) | 0.16 | 2.85 (2.15) | 0.66 |
| **Actinobacteria** | 2.98 (5.18) | 6.28 (6.81) | 0.28 | 2.04 (3.22) | 0.65 | 1.85 (1.88) | 0.52 |
| **Verrucomicrobia** | 1.00 (1.47) | 1.08 (3.24) | 0.95 | 0.23 (0.60) | 0.60 | 3.06 (6.47) | 0.10 |
| **PSC =** Primary Sclerosing Cholangitis; **UC =** Ulcerative Colitis; **PSC + UC** = Presence of  both diseases; *^a^* Significant when *P* ≤ 0.05; * Sidak’s post-hoc. | | | | | | | |
