# Supplementary material for: Gut Microbiome of Children and Adolescents With Primary Sclerosing Cholangitis in Association With Ulcerative Colitis
Source: Front Immunol. 2021 Feb 5;11:598152. doi: 10.3389/fimmu.2020.598152 (PMC7893080; doi:10.3389/fimmu.2020.598152)
Supplement: Supplementary file 4 [file Table_3.docx]

| **Supplementary Table 3**. Relative abundance of the main genera observed in controls and case groups. | | | | | | | |
| --- | --- | --- | --- | --- | --- | --- | --- |
| **Groups**  **Genera** | **Control** | **UC** | | **PSC + UC** | | **PSC** | |
|  | Mean (SD) | Mean (SD) | *P ^a^* | Mean (SD) | *P ^a^* | Mean (SD) | *P ^a^* |
| ***Bifidobacterium*** | 2.22 (5.27) | 4.39 (6.57) | 0.20 | 1.70 (3.30) | 0.80 | 0.95 (0.88) | 0.46 |
| ***Bacteroides*** | 18.70 (13.34) | 21.13 (12.13) | 0.62 | 14.50 (20.10) | 0.48 | 15.88 (13.82) | 0.58 |
| ***Parabacteroides*** | 1.35 (1.28) | 1.30 (1.71) | 0.92 | 1.93 (2.25) | 0.34 | 0.94 (0.73) | 0.43 |
| ***Prevotella 9*** | 6.92 (12.19) | 5.06 (13.80) | 0.70 | 16.30 (18.09) | 0.11 | 10.47 (14.19) | 0.47 |
| ***Alistipes*** | 2.14 (2.17) | 3.18 (3.26) | 0.36 | 2.64 (3.57) | 0.71 | 2.75 (4.50) | 0.59 |
| ***Lactobacillus*** | 2.20 (9.01) | 3.23 (4.72) | 0.71 | 2.19 (5.47) | 0.99 | 3.45 (9.88) | 0.66 |
| ***Streptococcus*** | 0.44 (0.64) | 0.53 (1.01) | 0.91 | 1.46 (1.93) | 0.30 | 2.55 (4.65) | 0.07 |
| ***Christensenellaceae R7 group*** | 2.17 (2.55) | 1.15 (1.19) | 0.12 | 0.20 (0.40) | 0.08 | 1.46 (1.55) | 0.30 |
| ***Lachnospiraceae NK4A136 group*** | 2.50 (2.58) | 1.77 (3.10) | 0.38 | 0.63 (0.88) | 0.06 | 1.33 (1.58) | 0.17 |
| ***Roseburia*** | 2.84 (5.03) | 1.33 (2.23) | 0.23 | 0.84 (0.97) | 0.19 | 2.30 (2.19) | 0.67 |
| ***Other Lachnospiraceas*** | 5.00 (4.22) | 4.07 (4.50) | 0.56 | 5.80 (7.84) | 0.69 | 4.15 (2.90) | 0.61 |
| ***Faecalibacterium*** | 1.45 (1.11) | 3.96 (7.63) | 0.08 | 1.63 (1.86) | 0.92 | 2.00 (3.43) | 0.71 |
| ***Ruminoclostridium 5*** | 1.65 (2.61) | 2.43 (2.86) | 0.34 | 0.27 (0.26) | 0.16 | 1.04 (1.69) | 0.46 |
| ***Ruminococcaceae UCG 002*** | 7.70 (6.40) | 4.95 (5.39) | 0.13 | 2.07 (2.09) | 0.06 | 4.99 (3.91) | 0.15 |
| ***Ruminococcus*** | 1.15 (1.10) | 0.72 (0.95) | 0.28 | 0.60 (1.17) | 0.25 | 0.95 (1.39) | 0.62 |
| ***Subdoligranulum*** | 3.76 (4.59) | 2.09 (3.78) | 0.18 | 0.91 (0.99) | 0.06 | 1.28 (1.63) | 0.28 |
| ***Eubacterium coprostanoligenes group*** | 4.06 (3.85) | 1.68 (2.67) | 0.22 | 1.47 (2.15) | 0.07 | 1.99 (3.58) | 0.08 |
| ***Non-cultivated Ruminococcaceas*** | 3.14 (3.34) | 3.20 (4.20) | 0.96 | 0.53 (0.43) | 0.08 | 2.00 (4.04) | 0.36 |
| ***Acidaminococcus*** | 0.11 (0.31) | 3.19 (7.41) | 0.17 | 2.11 (5.46) | 0.24 | 0.09 (0.30) | 0.99 |
| ***Phascolarctobacterium*** | 3.56 (4.85) | 1.71 (3.07) | 0.21 | 3.53 (3.73) | 0.98 | 3.55 (4.40) | 1.00 |
| ***Dialister*** | 1.66 (2.46) | 2.56 (4.18) | 0.42 | 1.04 (2.45) | 0.65 | 2.95 (3.73) | 0.26 |
| ***Megasphaera*** | 0.32 (1.33) | 0.13 (0.38) | 0.94 | 0.84 (1.97) | 0.87 | 6.51 (16.96) | 0.16 |
| ***Veillonella*** | 0.46 (0.61) | 2.42 (6.58) | 0.24 | 7.63 (7.77) | 0.002* | 2.95 (5.01) | 0.15 |
| ***Escherichia-Shigella*** | 3.11 (9.66) | 2.02 (5.01) | 0.67 | 3.17 (8.17) | 0.98 | 0.56 (0.71) | 0.33 |
| ***Akkermansia*** | 1.00 (1.47) | 1.08 (3.24) | 0.95 | 0.23 (0.60) | 0.60 | 3.05 (6.47) | 0.10 |
| **PSC =** Primary Sclerosing Cholangitis; **UC =** Ulcerative Colitis; **PSC + UC** = Presence of both diseases; *^a^* Significant when *P* ≤ 0.05; * Sidak’s post-hoc. | | | | | | | |
