# Supplementary material for: Gut Microbiome of Children and Adolescents With Primary Sclerosing Cholangitis in Association With Ulcerative Colitis
Source: Front Immunol. 2021 Feb 5;11:598152. doi: 10.3389/fimmu.2020.598152 (PMC7893080; doi:10.3389/fimmu.2020.598152)
Supplement: Supplementary file 5 [file Table_4.docx]

| **Supplementary Table 4.** Correlation between clinical data and bacterial genera. | | |
| --- | --- | --- |
| **Ulcerative Colitis** | ***Megasphaera*** | ***Veillonella*** |
| **Total Bilirubin (mg/dL)** Pearson’s r ^a^  *P*-value | 0.046 | 0.501 |
|  | 0.447 | 0.058 |
| **GGT (U/L)** Pearson’s r  ^a^  *P*-value | -0.266 | -0.474 |
|  | 0.201 | 0.060 |
| **Primary Sclerosing Cholangitis** | ***Megasphaera*** | ***Veillonella*** |
| **Total Bilirubin (mg/dL)** Pearson’s r  ^a^  *P*-value | -0.126 | 0.473 |
|  | 0.356 | 0.071 |
| **GGT (U/L)** Pearson’s r  ^a^  *P*-value | -0.229 | 0.018 |
|  | 0.249 | 0.479 |
| **PSC + UC** | ***Megasphaera*** | ***Veillonella*** |
| **Total Bilirubin (mg/dL)** Pearson’s r  ^a^  *P*-value | 0.026 | 0.800 |
|  | 0.478 | 0.015* |
| **GGT (U/L)** Pearson’s r  ^a^  *P*-value | 0.727 | 0.335 |
|  | 0.032* | 0.231 |
| ^a^ Pearson’s correlation (one-tailed); *Significant when  *P* ≤ 0.05.  **GGT =** Gamma-Glutamyl Transferase; **PSC + UC** = Presence of both diseases. | | |
