# Supplementary material for: Gut Microbiome of Children and Adolescents With Primary Sclerosing Cholangitis in Association With Ulcerative Colitis
Source: Front Immunol. 2021 Feb 5;11:598152. doi: 10.3389/fimmu.2020.598152 (PMC7893080; doi:10.3389/fimmu.2020.598152)
Supplement: Supplementary file 6 [file Table_5.docx]

| **Supplementary Table 5**. Relative abundance of the main phyla in controls and cases with < 10 years. | | | | | | | |
| --- | --- | --- | --- | --- | --- | --- | --- |
| **Groups**  **Phyla** | **Control** | **UC** | | **PSC + UC** | | **PSC** | |
|  | Mean (SD) | Mean (SD) | *P ^a^* | Mean (SD) | *P ^a^* | Mean (SD) | *P ^a^* |
| **Firmicutes** | 59.47 (18.52) | 48.92 (20.06) | 0.21 | 45.77 (18.17) | 0.21 | 63.03 (12.95) | 0.74 |
| **Bacteroidetes** | 34.31 (17.09) | 38.22 (16.96) | 0.64 | 29.80 (30.13) | 0.68 | 28.60 (9.16) | 0.60 |
| **Proteobacteria** | 1.91 (1.56) | 1.43 (1.29) | 0.92 | 20.57 (31.57) | 0.01* | 2.53 (2.58) | 0.92 |
| **Actinobacteria** | 1.07 (0.84) | 7.90 (8.95) | 0.01* | 3.63 (4.88) | 0.38 | 1.30 (0.61) | 0.94 |
| **Verrucomicrobia** | 0.75 (1.13) | 1.93 (4.59) | 0.37 | 0.00 | 0.65 | 2.93 (4.24) | 0.20 |
| **PSC =** Primary Sclerosing Cholangitis; **UC =** Ulcerative Colitis; **PSC + UC** = Presence of both diseases; *^a^* Significant when *P* ≤ 0.05; * Sidak’s post-hoc. | | | | | | | |
