# Supplementary material for: Gut Microbiome of Children and Adolescents With Primary Sclerosing Cholangitis in Association With Ulcerative Colitis
Source: Front Immunol. 2021 Feb 5;11:598152. doi: 10.3389/fimmu.2020.598152 (PMC7893080; doi:10.3389/fimmu.2020.598152)
Supplement: Supplementary file 7 [file Table_6.docx]

| **Supplementary Table 6**. Relative abundance of the main genera in controls and cases with < 10 years. | | | | | | | |
| --- | --- | --- | --- | --- | --- | --- | --- |
| **Groups**  **Genera** | **Control** | **UC** | | **PSC + UC** | | **PSC** | |
|  | Mean (SD) | Mean (SD) | *P ^a^* | Mean (SD) | *P ^a^* | Mean (SD) | *P ^a^* |
| ***Bifidobacterium*** | 0.48 (0.45) | 6.62 (8.95) | 0.04* | 3.13 (5.17) | 0.36 | 0.90 (0.53) | 0.88 |
| ***Bacteroides*** | 20.65 (12.82) | 21.13 (9.97) | 0.93 | 13.27 (14.71) | 0.31 | 15.07 (12.76) | 0.44 |
| ***Parabacteroides*** | 1.41 (1.44) | 0.98 (1.31) | 0.54 | 2.73 (2.50) | 0.14 | 0.93 (0.75) | 0.59 |
| ***Prevotella 9*** | 6.25 (8.70) | 8.32 (19.64) | 0.72 | 7.03 (6.37) | 0.91 | 8.67 (7.43) | 0.74 |
| ***Alistipes*** | 2.83 (2.42) | 2.97 (2.87) | 0.91 | 3.97 (4.24) | 0.48 | 1.13 (1.79) | 0.29 |
| ***Lactobacillus*** | 0.29 (0.41) | 2.93 (4.01) | 0.09 | 5.00 (8.31) | 0.11 | 0.70 (1.04) | 0.84 |
| ***Streptococcus*** | 0.53 (0.84) | 0.67 (1.40) | 0.92 | 1.73 (2.57) | 0.48 | 7.00 (7.99) | 0.001* |
| ***Christensenellaceae R7 group*** | 1.78 (1.98) | 1.17 (0.99) | 0.42 | 0.07 (0.06) | 0.08 | 2.07 (2.05) | 0.77 |
| ***Lachnospiraceae NK4A136 group*** | 2.23 (2.32) | 0.81 (1.26) | 0.12 | 0.33 (0.25) | 0.10 | 3.03 (2.22) | 0.48 |
| ***Roseburia*** | 3.86 (6.48) | 0.75 (1.09) | 0.16 | 1.10 (1.57) | 0.34 | 4.17 (2.85) | 0.92 |
| ***Other Lachnospiraceas*** | 5.01 (3.85) | 4.95 (6.13) | 0.98 | 2.43 (2.17) | 0.32 | 5.37 (2.70) | 0.89 |
| ***Faecalibacterium*** | 1.45 (1.27) | 6.82 (10.39) | 0.18 | 0.57 (0.51) | 0.78 | 1.13 (0.25) | 0.92 |
| ***Ruminoclostridium 5*** | 1.91 (2.77) | 3.22 (3.74) | 0.31 | 0.37 (0.32) | 0.35 | 0.87 (1.10) | 0.53 |
| ***Ruminococcaceae UCG 002*** | 7.35 (4.92) | 3.25 (2.41) | 0.22 | 2.23 (2.95) | 0.25 | 7.00 (5.91) | 0.89 |
| ***Ruminococcus*** | 1.56 (1.26) | 0.48 (0.39) | 0.19 | 1.10 (1.82) | 0.48 | 0.33 (0.32) | 0.06 |
| ***Subdoligranulum*** | 3.87 (5.55) | 2.85 (5.38) | 0.65 | 0.63 (1.01) | 0.27 | 1.67 (1.45) | 0.45 |
| ***Eubacterium coprostanoligenes group*** | 4.95 (4.50) | 0.93 (0.99) | 0.08 | 2.70 (3.08) | 0.28 | 2.20 (2.33) | 0.19 |
| ***Non-cultivated Ruminococcaceas*** | 3.73 (3.57) | 4.07 (5.22) | 0.84 | 0.70 (0.60) | 0.17 | 0.73 (0.57) | 0.18 |
| ***Acidaminococcus*** | 0.01 (0.03) | 2.72 (6.37) | 0.17 | 4.83 (8.37) | 0.06 | 0.00 | 0.99 |
| ***Phascolarctobacterium*** | 5.29 (5.61) | 1.10 (1.88) | 0.06 | 3.87 (4.63) | 0.61 | 3.10 (5.20) | 0.44 |
| ***Dialister*** | 1.15 (1.56) | 1.03 (1.26) | 0.90 | 0.10 (0.17) | 0.40 | 3.03 (5.17) | 0.14 |
| ***Megasphaera*** | 0.62 (1.83) | 0.00 | 0.35 | 0.10 (0.17) | 0.54 | 0.97 (1.67) | 0.68 |
| ***Veillonella*** | 0.76 (0.69) | 0.50 (0.95) | 0.87 | 5.70 (6.53) | 0.08 | 6.50 (7.94) | 0.03* |
| ***Escherichia-Shigella*** | 0.77 (1.26) | 0.43 (0.56) | 0.86 | 7.37 (12.41) | 0.04* | 0.30 (0.44) | 0.85 |
| ***Akkermansia*** | 0.75 (1.13) | 1.93 (4.59) | 0.37 | 0.00 | 0.66 | 2.93 (4.24) | 0.20 |
| **PSC =** Primary Sclerosing Cholangitis; **UC =** Ulcerative Colitis; **PSC + UC** = Presence of both diseases; *^a^* Significant when *P* ≤ 0.05; * Sidak’s post-hoc. | | | | | | | |
