# Supplementary material for: Gut Microbiome of Children and Adolescents With Primary Sclerosing Cholangitis in Association With Ulcerative Colitis
Source: Front Immunol. 2021 Feb 5;11:598152. doi: 10.3389/fimmu.2020.598152 (PMC7893080; doi:10.3389/fimmu.2020.598152)
Supplement: Supplementary file 8 [file Table_7.docx]

| **Supplementary Table 7**. Relative abundance of the main phyla in controls and cases with > 10 years. | | | | | | | |
| --- | --- | --- | --- | --- | --- | --- | --- |
| **Groups**  **Phyla** | **Control** | **UC** | | **PSC + UC** | | **PSC** | |
|  | Mean (SD) | Mean (SD) | *P ^a^* | Mean (SD) | *P ^a^* | Mean (SD) | *P ^a^* |
| **Firmicutes** | 54.86 (16.47) | 57.78 (15.29) | 0.37 | 42.45 (23.01) | 0.16 | 54.42 (21.09) | 0.11 |
| **Bacteroidetes** | 29.23 (17.18) | 30.62 (21.75) | 0.88 | 52.18 (23.71) | 0.18 | 35.28 (19.18) | 0.48 |
| **Proteobacteria** | 7.20 (14.14) | 6.17 (6.61) | 0.82 | 2.87 (2.70) | 0.39 | 2.96 (2.15) | 0.30 |
| **Actinobacteria** | 5.07 (7.03) | 4.65 (3.95) | 0.85 | 0.85 (0.70) | 0.11 | 2.05 (2.19) | 0.15 |
| **Verrucomicrobia** | 1.28 (1.80) | 0.22 (0.53) | 0.59 | 0.40 (0.80) | 0.70 | 3.11 (7.39) | 0.32 |
| **PSC =** Primary Sclerosing Cholangitis; **UC =** Ulcerative Colitis; **PSC + UC** = Presence of both diseases; *^a^* Significant when *P* ≤ 0.05; * Sidak’s post-hoc. | | | | | | | |
