# Supplementary material for: Gut Microbiome of Children and Adolescents With Primary Sclerosing Cholangitis in Association With Ulcerative Colitis
Source: Front Immunol. 2021 Feb 5;11:598152. doi: 10.3389/fimmu.2020.598152 (PMC7893080; doi:10.3389/fimmu.2020.598152)
Supplement: Supplementary file 9 [file Table_8.docx]

| **Supplementary Table 8**. Relative abundance of the main genera in controls and cases with > 10 years. | | | | | | | |
| --- | --- | --- | --- | --- | --- | --- | --- |
| **Groups**  **Genera** | **Control** | **UC** | | **PSC + UC** | | **PSC** | |
|  | Mean (SD) | Mean (SD) | *P ^a^* | Mean (SD) | *P ^a^* | Mean (SD) | *P ^a^* |
| ***Bifidobacterium*** | 4.14 (7.33) | 2.17 (1.74) | 0.37 | 0.62 (0.64) | 0.16 | 0.96 (1.01) | 0.11 |
| ***Bacteroides*** | 16.54 (14.25) | 21.13 (14.97) | 0.56 | 15.42 (25.71) | 0.90 | 16.19 (15.02) | 0.96 |
| ***Parabacteroides*** | 1.28 (1.14) | 1.62 (2.12) | 0.63 | 1.32 (2.19) | 0.96 | 0.94 (0.77) | 0.60 |
| ***Prevotella 9*** | 7.65 (15.65) | 1.80 (2.75) | 0.42 | 23.25 (21.85) | 0.06 | 11.15 (16.43) | 0.60 |
| ***Alistipes*** | 1.38 (1.67) | 3.38 (3.89) | 0.25 | 1.65 (3.23) | 0.89 | 3.36 (5.14) | 0.21 |
| ***Lactobacillus*** | 4.29 (13.07) | 3.53 (5.71) | 0.88 | 0.07 (0.10) | 0.46 | 4.49 (11.61) | 0.97 |
| ***Streptococcus*** | 0.34 (0.32) | 0.40 (0.51) | 0.88 | 1.25 (1.70) | 0.05 | 0.89 (1.00) | 0.15 |
| ***Christensenellaceae R7 group*** | 2.60 (3.11) | 1.13 (1.46) | 0.16 | 0.30 (0.53) | 0.05 | 1.24 (1.41) | 0.15 |
| ***Lachnospiraceae NK4A136 group*** | 2.79 (2.94) | 2.72 (4.16) | 0.95 | 0.85 (1.16) | 0.18 | 0.69 (0.66) | 0.07 |
| ***Roseburia*** | 1.72 (2.63) | 1.90 (2.99) | 0.87 | 0.65 (0.37) | 0.39 | 1.60 (1.58) | 0.90 |
| ***Other Lachnospiraceas*** | 5.00 (4.81) | 3.18 (2.27) | 0.45 | 8.32 (9.99) | 0.22 | 3.70 (3.02) | 0.55 |
| ***Faecalibacterium*** | 1.44 (0.99) | 1.10 (0.58) | 0.77 | 2.42 (2.18) | 0.45 | 2.32 (4.04) | 0.40 |
| ***Ruminoclostridium 5*** | 1.35 (2.52) | 1.63 (1.57) | 0.77 | 0.20 (0.22) | 0.29 | 1.10 (1.93) | 0.78 |
| ***Ruminococcaceae UCG 002*** | 8.08 (7.98) | 6.65 (7.15) | 0.63 | 1.95 (1.69) | 0.07 | 4.24 (3.08) | 0.15 |
| ***Ruminococcus*** | 0.69 (0.69) | 0.95 (1.30) | 0.63 | 0.22 (0.33) | 0.45 | 1.17 (1.58) | 0.33 |
| ***Subdoligranulum*** | 3.63 (3.54) | 1.33 (1.08) | 0.05 | 1.12 (1.07) | 0.06 | 1.14 (1.76) | 0.12 |
| ***Eubacterium coprostanoligenes group*** | 3.08 (2.90) | 2.43 (3.66) | 0.68 | 0.55 (0.56) | 0.16 | 1.91 (4.08) | 0.42 |
| ***Non-cultivated Ruminococcaceas*** | 2.49 (3.12) | 2.33 (3.14) | 0.92 | 0.40 (0.29) | 0.27 | 2.47 (4.72) | 0.99 |
| ***Acidaminococcus*** | 0.23 (0.43) | 3.67 (8.93) | 0.08 | 0.07 (0.15) | 0.94 | 0.12 (0.35) | 0.95 |
| ***Phascolarctobacterium*** | 1.65 (3.10) | 2.32 (4.04) | 0.71 | 3.27 (3.65) | 0.43 | 3.72 (4.45) | 0.21 |
| ***Dialister*** | 2.21 (3.17) | 4.08 (5.59) | 0.31 | 1.75 (3.24) | 0.83 | 2.92 (3.50) | 0.68 |
| ***Megasphaera*** | 0.00 | 0.27 (0.52) | 0.96 | 1.40 (2.60) | 0.81 | 8.59 (19.80) | 0.07 |
| ***Veillonella*** | 0.12 (0.22) | 4.33 (9.25) | 0.12 | 9.07 (9.26) | 0.02* | 1.61 (3.23) | 0.55 |
| ***Escherichia-Shigella*** | 5.69 (13.83) | 3.60 (6.98) | 0.63 | 0.02 (0.05) | 0.25 | 0.66 (0.79) | 0.21 |
| ***Akkermansia*** | 1.28 (1.80) | 0.22 (0.53) | 0.59 | 0.40 (0.80) | 0.70 | 3.10 (7.40) | 0.32 |
| **PSC =** Primary Sclerosing Cholangitis; **UC =** Ulcerative Colitis; **PSC + UC** = Presence of both diseases; *^a^* Significant when *P* ≤ 0.05; * Sidak’s post-hoc. | | | | | | | |
