# Supplementary material for: Gut Microbiome of Children and Adolescents With Primary Sclerosing Cholangitis in Association With Ulcerative Colitis
Source: Front Immunol. 2021 Feb 5;11:598152. doi: 10.3389/fimmu.2020.598152 (PMC7893080; doi:10.3389/fimmu.2020.598152)
Supplement: Supplementary file 10 [file Table_9.docx]

| **Supplementary Table 9**. Alpha diversity indices in controls and cases with < 10 years. | | | | | | | |
| --- | --- | --- | --- | --- | --- | --- | --- |
| **Groups**  **Indices** | **Control** | **UC** | | **PSC + UC** | | **PSC** | |
|  | Mean  (SD) | Mean (SD) | *P ^a^* | Mean (SD) | *P ^a^* | Mean (SD) | *P ^a^* |
| **Chao1** | 4416.07 (3230.94) | 3589.26 (2006.99) | 0.42 | 2347.90 (733.04) | 0.05 | 3618.83 (938.32) | 0.55 |
| **Shannon** | 4.88 (0.52) | 4.44 (0.73) | 0.11 | 4.31 (0.56) | 0.10 | 4.86 (0.62) | 0.94 |
| **Simpson** | 0.96  (0.02) | 0.94 (0.03) | 0.11 | 0.95 (0.03) | 0.35 | 0.97 (0.01) | 0.89 |
| **Observed OTUs** | 2759.45 (2657.18) | 2332.33 (1906.75) | 0.61 | 1292.67 (86.00) | 0.07 | 1999.00 (419.73) | 0.44 |
| **PSC =** Primary Sclerosing Cholangitis; **UC =** Ulcerative Colitis; **PSC + UC** = Presence of both diseases; *^a^* Significant when *P* ≤ 0.05; * Sidak’s post-hoc. | | | | | | | |
