# Supplementary material for: Gut Microbiome of Children and Adolescents With Primary Sclerosing Cholangitis in Association With Ulcerative Colitis
Source: Front Immunol. 2021 Feb 5;11:598152. doi: 10.3389/fimmu.2020.598152 (PMC7893080; doi:10.3389/fimmu.2020.598152)
Supplement: Supplementary file 11 [file Table_10.docx]

| **Supplementary Table 10**. Alpha diversity indices in controls and cases with > 10 years. | | | | | | | |
| --- | --- | --- | --- | --- | --- | --- | --- |
| **Groups**  **Indices** | **Control** | **UC** | | **PSC + UC** | | **PSC** | |
|  | Mean  (SD) | Mean (SD) | *P ^a^* | Mean  (SD) | *P ^a^* | Mean (SD) | *P ^a^* |
| **Chao1** | 3509.36 (1433.62) | 2619.20 (859.83) | 0.11 | 2828.62 (1252.03) | 0.30 | 2847.36 (972.31) | 0.21 |
| **Shannon** | 4.64 (0.75) | 4.62 (0.52) | 0.96 | 4.65  (0.25) | 0.98 | 4.39 (0.84) | 0.43 |
| **Simpson** | 0.94  (0.05) | 0.96 (0.02) | 0.52 | 0.96  (0.01) | 0.47 | 0.91 (0.09) | 0.45 |
| **Observed OTUs** | 2082.70 (1088.79) | 1391.00 (340.12) | 0.13 | 1661.50 (663.47) | 0.28 | 1519.25 (384.80) | 0.06 |
| **PSC =** Primary Sclerosing Cholangitis; **UC =** Ulcerative Colitis; **PSC + UC** = Presence of both diseases; *^a^* Significant when *P* ≤ 0.05; * Sidak’s post-hoc. | | | | | | | |
