# Supplementary material for: Gut Microbiome of Children and Adolescents With Primary Sclerosing Cholangitis in Association With Ulcerative Colitis
Source: Front Immunol. 2021 Feb 5;11:598152. doi: 10.3389/fimmu.2020.598152 (PMC7893080; doi:10.3389/fimmu.2020.598152)
Supplement: Supplementary file 12 [file Table_11.docx]

| **Supplementary Table 11**. Relative abundance of the main genera according to the disease state. | | | |
| --- | --- | --- | --- |
| **Disease State**  **Genera** | **Activity** | **Remission**^b^ **/ Controlled**^c^ | *P ^a^* |
|  | Mean (SD) | Mean (SD) |  |
| ***Bifidobacterium*** | 1.57 (1.30) | 3.12 (1.06) | 0.35 |
| ***Prevotella 9*** | 11.16 (4.28) | 8.67 (3.49) | 0.65 |
| ***Lactobacillus*** | 0.77 (0.85) | 1.97 (0.70) | 0.28 |
| ***Ruminoclostridium 5*** | 0.37 (0.58) | 2.10 (0.47) | 0.02* |
| ***Ruminococcaceae UCG 002*** | 2.52 (1.15) | 5.48 (0.94) | 0.047* |
| ***Veillonella*** | 7.06 (1.68) | 1.67 (1.37) | 0.01* |
| ***Escherichia-Shigella*** | 3.65 (1.33) | 0.49 (1.09) | 0.06 |
| ***Akkermansia*** | 0.42 (1.23) | 2.39 (1.01) | 0.21 |
| *^a^* Significant when *P*≤ 0.05; * Sidak’s post-hoc; ^b^ Patients in the UC or PSC + UC group;  ^c^ Patients in the PSC group. | | | |
